# State of the Art of Aortic Valve Reimplantation for Aortic Root Aneurysms

**DOI:** 10.21470/1678-9741-2026-0070

**Published:** 2026-11-01

**Authors:** Tirone E. David

**Affiliations:** 1 Division of Cardiovascular Surgery, Toronto General Hospital, Peter Munk Cardiac Centre, University Health Network, University of Toronto, Toronto, Ontario, Canada

**Keywords:** Aortic Operation (Operation on the aorta), Aortic Root, Aortic Valve, Repair.

## Abstract

This article describes the development of aortic valve-sparing operations and their current status in surgical practice. They are classified into two general types of operations: aortic root remodeling and aortic valve reimplantation. Both techniques have been modified over the years from their original descriptions, but both remain useful in appropriately selected patients with aortic root and proximal aortic aneurysms, provided the aortic valve cusps are reasonable. Aortic valve reimplantation has proven more durable in young patients with aortic root aneurysms associated with genetic syndromes, whereas remodeling of the aortic root may be appropriate for older patients with ascending aortic aneurysms, dilated sinotubular junction, and secondarily dilated aortic sinuses. These operations were developed to preserve the native aortic valve and have expanded the armamentarium to treat patients with aortic root aneurysms and reasonably normal aortic cusps. In addition to a description of their development, a summary of current results of aortic valve reimplantation worldwide is presented, along with the author’s personal perspective on its reproducibility.

## DEVELOPMENT OF AORTIC VALVE-SPARING OPERATIONS

**Table:** 

Abbreviations, Acronyms & Symbols	
AI	= Aortic insufficiency
BAV	= Bicuspid aortic valves
CI	= Confidence interval
DIS	= Aortic dissection
NA	= Not available
STJ	= Sinotubular junction
TEE	= Transesophageal echocardiography

Most innovative operative procedures result from a stepwise, logical progression of knowledge and experience from previously known operative procedures. The development of aortic valve-sparing operations was no exception. Early in my career as an academic cardiac surgeon, I reintroduced stentless porcine bioprosthetic aortic valves to clinical practice, aiming to improve their hemodynamic performance and durability. Implanting stentless bioprosthetic aortic valves required greater knowledge of the functional anatomy of the aortic valve, which forced me to learn the interplay among the aortic root components that control aortic valve function, that is, the aortic cusps, aortic annulus, and sinotubular junction (STJ). We learned that dilatation of the STJ can cause aortic insufficiency (AI) by pulling the cusps apart, preventing them from sealing the aortic valve orifice. Yet the aortic cusps may remain normal or show only mild stretching of their free margins in patients with aortic root and ascending aortic aneurysms. Thus, patients with ascending aortic aneurysm and AI were treated by simple replacement of the ascending aorta with a tubular Dacron® graft and restoring the normal diameter of the SJT^[1]^. The next step in the learning curve was to replace the ascending aorta and one abnormal aortic sinus with a properly tailored tubular Dacron®, and from there, to replace all three aortic sinuses with a single correctly tailored tubular graft. The operative procedures described above were a stepwise progression of acquired new knowledge and experience. I was unaware that Sir Magdi Yacoub had performed similar operations as early as 1979 because he did not report it until 1993^[2]^.

Intraoperative AI after aortic valve-preserving operations in those days was estimated after aortic unclamping by observing the arterial tracing with the heart empty and beating on total cardiopulmonary bypass. If the ventricle was not ejecting and the ventricular vent return was < 100 ml/min, we assumed that the valve was competent. This all changed in my practice in 1988, when transesophageal echocardiography (TEE) became available. Its impact on valve repair surgery was dramatic. Not only could we determine whether AI was present at the end of the operation, but with experience, we also learned the reason for postoperative AI. Aortic cusp prolapse was the most common cause of AI after those operations.

TEE also identified annular dilatation as a potential cause of AI after surgery. Annuloaortic ectasia is common in young patients with aortic root aneurysms, and adjusting the STJ diameter and correcting cusp prolapse are often inadequate to achieve aortic valve competence. That prompted us to develop an aortic annuloplasty by suturing a strip of Dacron® fabric on the outside of the aortic root along its fibrous components^[1]^.

By that time, an aortic valve-preserving operation in young patients with dilated aortic annulus had become more complicated: the aortic sinuses and coronary arteries had to be detached from the native aortic root, the aortic root had to be dissected to a level below the aortic annulus to allow placement of a Dacron® band to reduce the diameter of the aortic annulus, the aortic sinuses recreated with a tailored Dacron® graft, cusp prolapse had to be corrected, and the coronary arteries reimplanted. In addition, bleeding along all suture lines in the reconstructed aortic root was a serious problem in some of the patients.

In the summer of 1989, through a stepwise, logical progression of knowledge and experience, the concept of reimplantation of the aortic valve emerged^[3]^. Instead of an external annuloplasty band to reduce the diameter of the aortic annulus, I secured a tubular Dacron® graft on the outside of the aortic root, at the level of the left ventricular outflow tract, which corrected the dilated aortic annulus. Resuspension of the commissures within the graft was relatively straightforward owing to my experience with stentless aortic valves. Estimating cusp prolapse became simpler because the commissures were suspended into a relatively rigid structure, and the level of coaptation of the three cusps was easy to determine. Thus, a new type of aortic valve-preserving operation was developed. To distinguish these operations from aortic valve repair, we introduced the term aortic valve-sparing operations and classified them as either aortic root remodeling or aortic valve reimplantation^[4]^.

Aortic valve reimplantation into a tubular Dacron® graft has drawn considerable criticism because it eliminates the aortic sinuses, which regulate the opening and closure of the aortic cusps during the cardiac cycle. It is well known that normally the aortic valve cusps open and close much faster than the mitral valve leaflets. Placing the aortic valve inside a tubular Dacron® makes the cusps open and close even faster. This alteration is less evident after aortic root remodeling^[5]^. This knowledge prompted us to use grafts 2 or 4 mm larger than estimated for the aortic valve reimplantation. If the aortic annulus diameter was smaller than the graft, one of its ends was plicated in three areas corresponding to the lowest part of the aortic annulus to reduce the diameter of the graft. After reimplanting the aortic valve, the central portion of the intercommissural spaces was plicated at the level of the new STJ to create neo-aortic sinuses. Sometimes a second graft, 2 - 4 mm smaller, was sutured to the reconstructed aortic root at the level of the new STJ. This latter approach facilitates resuspension of the commissures and suturing of the aortic annulus within the graft. The creation of neo-aortic sinuses reduces the speed of cusp opening and closing, but it does not return to normal, likely because the Dacron® graft is noncompliant. In addition, it may adversely affect ventricular-aortic coupling.

I have never used commercially available Valsalva grafts because they are geometrically incorrect. Anatomic studies of the aortic root have shown that the aortic annulus is attached to a cylindrical structure and along a single horizontal plane, and the aortic sinuses are bulges in that cylindrical structure^[6]^. Commercially available Valsalva grafts have spherical sinuses, and if the aortic annulus is sutured inside the sphere, it may distort the annulus, which in turn may adversely affect the durability of the aortic cusps inside a rigid and anatomically incorrect structure.

Aortic valve reimplantation is also known as the David operation, and the aortic root remodeling as Yacoub operation.

## CLINICAL EXPERIENCE WITH AORTIC VALVE REIMPLANTATION AT TORONTO GENERAL HOSPITAL

From August 3, 1989, to December 31, 2019, aortic valve reimplantation was performed in 465 patients with a median age of 46 years^[7]^. There were 165 patients with Marfan syndrome, 12 with Loeys-Dietz syndrome, and 33 with acute or chronic type A dissections. Two-thirds of the patients had moderate AI. Patients have been followed prospectively with periodic echocardiographic assessment of aortic valve function and, when appropriate, computed tomography and magnetic resonance imaging of the residual aorta. The median follow-up was 10 years, and 25 patients were alive and without aortic valve reintervention at 20 years. The operations were performed by three attending surgeons, and the current operative technique has been described in detail in a recent publication^[8]^. The size of the graft is estimated by the average height of the cusps as originally described^[3]^. Approximately two-thirds of patients had the valve implanted into a straight tubular Dacron®, and the remaining one-third had the valve implanted in a graft 2 to 3 mm larger than estimated and plicated at the subannular level if its diameter was larger than the aortic annulus, and after reimplanting the aortic valve inside the graft, the intercommissural distance was reduced by plication of the graft at the level of the new STJ to create neo-aortic sinuses. Cusps with fenestrations close to the commissures were reinforced with a double layer of a fine expanded polytetrafluoroethylene suture^[9]^. Prolapsing cusps were shortened by plication of the free margins along the nodule of Arantius^[8,9]^. Other procedures were performed as needed, mitral valve repair being the most common.

The detailed clinical characteristics of these patients have been published previously^[7]^. In this cohort of 465 consecutive patients, there were five operative and 51 late deaths. Only age by five-year increments was associated with late mortality by multivariate analysis. The Kaplan-Meier estimate of patients’ survival was 75.2% at 20 years.

Reinterventions in the aortic valve were performed in 15 patients, from two days to 23 years after surgery. One patient had aortic valve repair for a cusp perforation, and 14 had aortic valve replacement (13 surgical and one transcatheter). The cumulative incidence of aortic valve reinterventions was 6% at 20 years. The number of reoperations was too small for meaningful multivariate analysis of risk factors associated with it.

Moderate or severe AI developed in 28 patients, ranging from two days to 27 years after surgery. The cumulative incidence of AI was 10.2% at 20 years. We could not identify variables associated with AI development by multivariable analysis.

We have also reported the outcomes of aortic valve-sparing operations in patients with Marfan syndrome^[10]^. We have documented that aortic valve reimplantation provides better long-term aortic valve function than aortic root remodeling, because the aortic annulus and subaortic connective tissues continue to dilate after surgery in some patients who had remodeling^[10]^. The cumulative incidence of moderate and severe AI at 20 years was 8.5% (95% confidence interval [CI]: 4.9%, 14.8%) after aortic valve reimplantation and 35.4% (95% CI 21.0%, 62.1%)^[10]^.

Finally, a word about patient selection. During the first five years of our experience, we performed aortic valve reimplantation only in patients with normal aortic cusps. As we became more confident with the procedure, we expanded it to patients with cusp prolapse, and in 1994, we introduced expanded polytetrafluoroethylene sutures to repair cusps with fenestrations along the commissures. The suture was passed in and out of the cusp tissue 1 mm below the free margin, from commissure to commissure. A second layer was applied slightly below the first and along the lower rim of the fenestrated cusp^[8,9]^. The long-term results of this procedure have had minimal adverse effect on the development of postoperative AI^[9]^.

We did not perform aortic valve reimplantation in patients with bicuspid aortic valves (BAV) and aortic root aneurysms until a decade into the experience. Patients with BAV and severe AI may have a grossly dilated aortic annulus without an aortic root aneurysm. After de Kerchove et al.^[11]^ showed that aortic valve reimplantation provides superior results compared with aortic valve repair in patients with BAV with severe AI and a dilated aortic annulus, we adopted their approach as another indication for aortic valve reimplantation. We do not have sufficient patient numbers to compare outcomes as de Kerchove et al.^[11]^. However, BAV includes a heterogeneous group of anatomic arrangements and commissural orientations that play an important role in the long-term outcome. Aortic valve reimplantation in patients with BAV has not resulted in outcomes as good as those with the tricuspid aortic valve in our experience^[7,12]^. However, we believe that the results of aortic valve reimplantation in patients with BAV and commissures close to 180° apart are likely to be as good as with a tricuspid aortic valve, but other orientations may not be as durable.

## CLINICAL OUTCOMES OF AORTIC VALVE REIMPLANTATION IN OTHER CENTERS

The late John Kirklin, the surgeon who named aortic valve reimplantation as “David operation”, asked me whether it was a reproducible operation and went on to tell me that he believed that “only reproducible operations are good operations”. Reports on the long-term outcomes of this operation have been variable, with some centers reporting better results than others.

Cardiac surgeons at Hannover Medical School (Hannover, Germany) adopted aortic valve reimplantation soon after its introduction to the scientific community at an American Association for Thoracic Surgery annual meeting, and they have not changed the technique since, that is, they continue using a tubular Dacron® graft^[13,14]^. One of the most significant contributions of the Hannover Group was the echocardiographic identification of the aortic coaptation level within the Dacron® graft as a predictor of failure^[13]^. That report prompted further investigation, and we learned that the aortic cusps must coat within the graft and at least 8 mm above the level of the aortic annulus. In addition, they probably have the largest number of patients ever reported to have undergone a single technique: from 1993 to 2019, 677 patients underwent aortic valve reimplantation with a straight tubular Dacron® graft for a variety of aortic root pathologies^[14]^. Freedom from valve-related reoperation was 80% at 20 years, but only 50% of patients were free from moderate or severe AI^[14]^.

Gebrine El Khoury, a surgeon from Brussels, Belgium, was also an early adopter of aortic valve-sparing operations. A gifted technician, he has contributed more to the dissemination of aortic valve reimplantation than anyone I know. He developed a systematic, didactic approach to analyzing the aortic root and the various steps of aortic valve reimplantation, which has helped numerous surgeons worldwide. He uses the height of the commissure between the left and non-coronary cusps to determine the size of the graft for aortic valve reimplantation instead of the aortic cusp heights. Moreover, he was an early adopter of commercially available Valsalva grafts. From 1988 through 2022, at his service, they performed 652 aortic valve reimplantation operations: 382 for tricuspid aortic valve, 263 for BAV, and five unicuspid or quadricuspid valves^[15]^. Unique to their experience is that 150 of those patients had no aortic root aneurysm, and the operation was performed to correct AI. Freedom from reoperation on the aortic valve at 12 years was 90.4%, with no difference across subgroups^[15]^.

Table 1 summarizes the long-term results of aortic valve reimplantation from several centres worldwide. It may be inappropriate to compare results due to heterogeneity in patients' clinical profiles and differences in operative techniques. More importantly, the proportion of patients who underwent periodic echocardiographic assessment of aortic valve function after surgery is small and highly inconsistent across reports. Freedom from reoperation is not equivalent to freedom from failure, that is, AI, because this lesion is often well tolerated when it develops insidiously, and the ventricle adapts to it. It is, however, a silent killer of myocytes.

**Table 1:** Summary of various reports on the long-term outcomes of aortic valve reimplantation.

Investigator	Number of Patients	Mean Age (years)	Genetic Syndrome	DIS	BAV	Follow-up (years)	Free from Reoperation			Free from AI		
David^[7]^	465	46	38%	7%	14%	10	99%	98%	96%	96%	94.3%	92.7%
Shrestha^[14]^	677	56	16%	20%	10%	10.4	92%	87%	84%	NA	NA	NA
Kubo^[16]^	363	51	21%	-	19%	6.4	92.8%	85.1%	78.3%	82.5%	85.1%	64%
Forteza Gil^[17]^	157	35	100%	4%	-	7.3	96%	96%	96%	91.8%	89.6%	73.3%
Tamer^[18]^	303	53	13.8%	4%	0	6.1	95%	90%	NA	81.2%	70.4%	NA
Chirichilli^[19]^	265	55	12%	3%	19%	7.0	97.8%	95.9%	95.9%	94.1%	92.2%	92.2%
Patlolla^[20]^	342	48	38%	14%	5.5%	8.9	91.6%	87.2%	82.9%	93%	92%	NA
Manganiello^[21]^	300	53	7%	3%	19%	7.0	97.1%	92.9%	92.5%	84.8%	74.3%	NA

AI=aortic insufficiency; BAV=bicuspid aortic valves; DIS=aortic dissection; NA=not available

## CONCLUSION

Aortic valve reimplantation is a complex operation, and it may not be easily reproducible. Unlike mitral valve repair, where some pathologies are easier to correct than others, aortic root aneurysms pose a more formidable challenge to preserve the aortic valve. The operative outcome of aortic valve reimplantation is affected by the size and type of Dacron® graft used, how the graft is secured to the left ventricular outflow tract, the spatial positioning of the commissures within the graft, the level where the cusps coapt, and the STJ. In addition to better-than-average technical skills, a sound knowledge of the functional anatomy of the aortic root is needed for the performance of this operation. However, when performed correctly, the long-term results are excellent and lead to an event-free life for several decades. Aortic valve reimplantation has been particularly important to young patients with aortic root aneurysms associated with a genetic syndrome.

Aortic valve-sparing operations expanded the surgical armamentarium for treating patients with aortic root aneurysms and improved their quality of life; however, much remains to be learned about their reproducibility and long-term outcomes.

## Data Availability

The author declares that the database is privately owned by his clinic and therefore cannot be shared publicly.

## Artificial Intelligence Usage

The author declares that no artificial intelligence tool was used in the preparation of this article.

## References

[1] David TE. (1996). Remodeling the aortic root and preservation of the native aortic
valve. Oper Tech Card Thorac Surg.

[2] Sarsam MA, Yacoub M. (1993). Remodeling of the aortic valve anulus. J Thorac Cardiovasc Surg.

[3] David TE, Feindel CM. (1992). An aortic valve-sparing operation for patients with aortic
incompetence and aneurysm of the ascending aorta. J Thorac Cardiovasc Surg.

[4] David TE, Feindel CM, Bos J. (1995). Repair of the aortic valve in patients with aortic insufficiency
and aortic root aneurysm. J Thorac Cardiovasc Surg.

[5] Leyh RG, Schmidtke C, Sievers HH, Yacoub MH. (1999). Opening and closing characteristics of the aortic valve after
different types of valve-preserving surgery. Circulation.

[6] Thubrikar M. (1989). The aortic valve.

[7] David TE, David CM, Ouzounian M, Feindel CM, Lafreniere-Roula M. (2021). A progress report on reimplantation of the aortic
valve. J Thorac Cardiovasc Surg.

[8] David T. (2021). Reimplantation valve-sparing aortic root replacement is the most
durable approach to facilitate aortic valve repair. JTCVS Tech.

[9] David TE, Seidman MA, David CM, Lafreniere-Roula M. (2023). Outcomes of reimplantation of the aortic valve in patients with
aortic cusp fenestration. Ann Thorac Surg.

[10] David TE, Park J, Tatangelo M, Steve Fan CP, Ouzounian M. (2023). Cardiovascular events after aortic root repair in patients with
Marfan syndrome. J Am Coll Cardiol.

[11] de Kerchove L, Boodhwani M, Glineur D, Vandyck M, Vanoverschelde JL, Noirhomme P (2011). Valve sparing-root replacement with the reimplantation technique
to increase the durability of bicuspid aortic valve repair. J Thorac Cardiovasc Surg.

[12] Ouzounian M, Feindel CM, Manlhiot C, David C, David TE. (2019). Valve-sparing root replacement in patients with bicuspid versus
tricuspid aortic valves. J Thorac Cardiovasc Surg.

[13] Pethig K, Milz A, Hagl C, Harringer W, Haverich A. (2002). Aortic valve reimplantation in ascending aortic aneurysm: risk
factors for early valve failure. Ann Thorac Surg.

[14] Shrestha M, Boethig D, Krüger H, Kaufeld T, Martens A, Haverich A (2023). Valve-sparing aortic root replacement using a straight tube graft
(David I procedure). J Thorac Cardiovasc Surg.

[15] Jahanyar J, de Kerchove L, Arabkhani B, Tsai PI, Aphram G, Mastrobuoni S (2023). Three decades of reimplantation of the aortic valve-the Brussels
experience. Ann Cardiothorac Surg.

[16] Kubo S, Tanaka A, Omura A, Tsunemi K, Oka T, Okada K (2024). Long-term results of valve-sparing aortic root replacement and
aortic cusp repair. Ann Thorac Surg.

[17] Forteza Gil A, Martinez-Lopez D, Centeno J, Rivas Oyarzabal J, García Suarez J, de Villarreal Soto JE (2022). Aortic valve reimplantation in patients with connective tissue
syndromes: a 15-year follow-up. Eur J Cardiothorac Surg.

[18] Tamer S, Mastrobuoni S, Lemaire G, Jahanyar J, Navarra E, Poncelet A (2021). Two decades of valve-sparing root reimplantation in tricuspid
aortic valve: impact of aortic regurgitation and cusp repair. Eur J Cardiothorac Surg.

[19] Chirichilli I, Scaffa R, Irace FG, Salica A, Weltert LP, D'Aleo S (2023). Twenty-year experience of aortic valve reimplantation using the
Valsalva graft. Eur J Cardiothorac Surg.

[20] Patlolla SH, Saran N, Dearani JA, Stulak JM, Schaff HV, Greason KL (2022). Outcomes and risk factors of late failure of valve-sparing aortic
root replacement. J Thorac Cardiovasc Surg.

[21] Manganiello S, Soquet J, Mugnier A, Rousse N, Juthier F, Banfi C (2023). David procedure: a 21-year experience with 300
patients. Ann Thorac Surg.

